# NF-kB as a key player in regulation of cellular radiation responses and identification of radiation countermeasures

**DOI:** 10.15190/d.2015.27

**Published:** 2015-03-31

**Authors:** Vijay Singh, Damodar Gupta, Rajesh Arora

**Affiliations:** Division of Radiation Biosciences, Institute of Nuclear Medicine & Allied Sciences, Brig SK Mazumdar Marg, Timarpur, Delhi, India

**Keywords:** Ionizing radiation, NF-kB, apoptosis, cell proliferation, inflammation, radioprotector

## Abstract

Nuclear factor (NF)-κB is a transcription factor that plays significant role in immunity, cellular survival and inhibition of apoptosis, through the induction of genetic networks. Depending on the stimulus and the cell type, the members of NF-κB related family (RelA, c-Rel, RelB, p50, and p52), forms different combinations of homo and hetero-dimers. The activated complexes (Es) translocate into the nucleus and bind to the 10bp κB site of promoter region of target genes in stimulus specific manner. In response to radiation, NF-κB is known to reduce cell death by promoting the expression of anti-apoptotic proteins and activation of cellular antioxidant defense system. Constitutive activation of NF-κB associated genes in tumour cells are known to enhance radiation resistance, whereas deletion in mice results in hypersensitivity to IR-induced GI damage. NF-κB is also known to regulate the production of a wide variety of cytokines and chemokines, which contribute in enhancing cell proliferation and tissue regeneration in various organs, such as the GI crypts stem cells, bone marrow etc., following exposure to IR. Several other cytokines are also known to exert potent pro-inflammatory effects that may contribute to the increase of tissue damage following exposure to ionizing radiation. Till date there are a series of molecules or group of compounds that have been evaluated for their radio-protective potential, and very few have reached clinical trials. The failure or less success of identified agents in humans could be due to their reduced radiation protection efficacy. In this review we have considered activation of NF-κB as a potential marker in screening of radiation countermeasure agents (RCAs) and cellular radiation responses. Moreover, we have also focused on associated mechanisms of activation of NF-κB signaling and their specified family member activation with respect to stimuli. Furthermore, we have categorized their regulated gene expressions and their function in radiation response or modulation. In addition, we have discussed some recently developed radiation countermeasures in relation to NF-κB activation

## SUMMARY

1. Introduction
2. NF-κB/IκB family members & their associated proteins
3. NF-κB activation pathways
  - 3.1 IKKβ dependent (classical) pathway
  - 3.2. IKKα dependent (alternative) pathway
  - 3.3. Atypical pathway
  - 3.4. Oxidative stress-induced pathway
4. Post translational modifications of NF-κB proteins
5. NF-κB regulated proteins and their functions in oxidative stress
6. Radiation Countermeasures in relation to NF-κB activation
7. Conclusion

## 1. Introduction

Deleterious effects of ionizing radiation (IR) may lead to significant morbidity and a possible fatal illness that affects various organs of the organism in a dose and time dependent manner^1^. Exposure of the organism to IR during therapy, or as a result of a radiological/ nuclear incident, or act of terrorism, may symbolize serious health issues. However, this problem remains largely impervious to medical management of IR exposure and therefore, there is a pressing need to develop safe and effective radiation countermeasures agents (RCA) to reduce or mitigate the harmful consequences of IR exposure at cellular, tissue and organism levels. Following exposure of the organism to ionizing radiation, various signaling pathways, such as the mitogen-activated protein kinase (MAPK), phosphoinositide 3-kinases (PI3K), and ataxia telangiectasia mutated (ATM) are activated and all these processes are tightly regulated in relation to changes in expression of various transcription factors (AP, NF-κB, p53, ARE, GADD153 etc) along with changes in the functional status of cell organelles^2-4^. This may trigger alterations in expression of a large number of genes that are mostly related to cell cycle progression, cell survival, DNA repair and apoptosis^4,5^.

**Figure 1:**
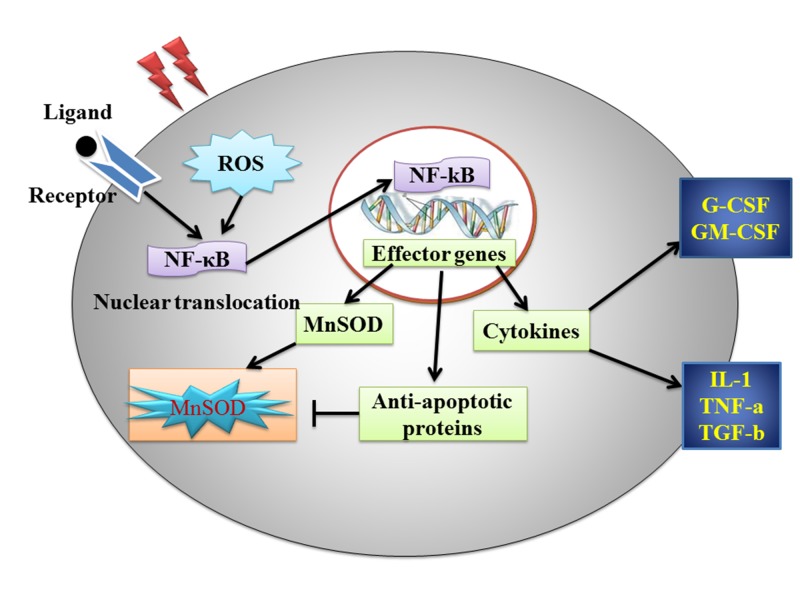
Schematic picture of nuclear factor (NF)-κB signaling events that influence the cellular responses to IR

NF-κB was first discovered by Baltimore & Sen as a B cell specific nuclear protein that binds to a site in the immunoglobulin kappa (Igk) light chain gene enhancer^6^. NF-κB is basically a highly conserved and inducible transcription factor, which regulates the expression of over 200 genes involved in a broad range of events, including the immune response^7^, inflammation^8^, differentiation, proliferation, cell survival, apoptosis^9,10^. The role of NF-κB in protection of cells from the complement dependent cytotoxicity has been recently reported by Gancz et al^11^. Although there are few exceptions where NF-κB contributes to cell death^12^, in most cases, the expression of NF-κB target genes promotes cellular survival. Normally, NF-κB transcription factor is bound to the Inhibitor(s) of kappa B (IκB) and is located in the cytoplasm. The NF-κB is activated by numerous stimuli through a variety of receptors or other intrinsic activation pathways. This recruits unique combinations of scaffolding and signaling proteins, that ultimately converge to the IκB kinase (IKK) complex. There are over 150 different stimuli that can activate NF-κB^13^. Most of the disparate ligands act upon similar cell surface and intracellular receptors including the cytokines (TNF-α, IL-1α/β and TRAIL)^14^, bacterial molecules (LPS, flagellin, and non-methylated dsDNA)^15^, viral components (dsDNA, dsRNA and ssRNA), DNA damaging agents (ionizing radiation or oxidative stress and chemotherapeutic drugs)^16,17^. A majority of NF-κB activators are functionally related to either pathogenic cellular invasion or a cellular insult that initiates an immune response. Overall, NF-κB is activated in parallel with other mitogenic pathways, through induction of its genetic network (**Figure 1**).

Abnormal activation of NF-κB subsidizes in many human diseases, such as in cancer and inflammatory diseases. Hence, elucidating how NF-κB signaling is regulated in different contexts is important for the identification and development of therapeutics for various ailments, such as atherosclerosis, asthma, arthritis and cancer^18,19^. NF-κB is one of the major targets for the screening and identification of promising radiation countermeasure agents (RCAs). In this review, we have mainly focused on NF-kB modulation following IR exposure and associated target genes for NF-kB in relation to identification of RCAs. We have also discussed the current status of RCAs, specifically their role in NF-κB activation.

## 2. NF-κB/IκB family members & associated proteins

**Figure 2:**
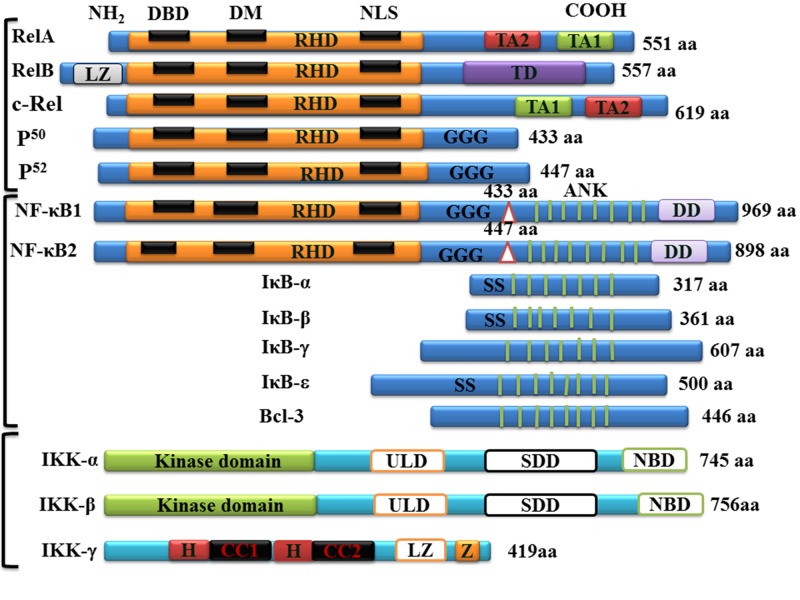
Schematic drawings of NF-κB/Rel proteins. Structures of the mammalian NF-κB, IκB, and IKK proteins The number of amino acids in each protein is indicated on the right. Presumed sites of cleavage for p105/NF-κB1 (amino acid 433) and p100/NF-κB2 (amino acid 447) are shown on the top of each protein. The positions of functional domains are indicated, including the Rel homology domain (RHD), DNA binding domain (DBD), dimerization domain (DM), nuclear localization signal (NLS), transactivation domains (TD). TA1 and TA2 subdomain of TD presented in RelA and cRel, glycine-rich hinge region (GGG), ankyrin repeats (ANK), double serine phosphorylation sites (SS), leucine zipper (LZ), helix-loop-helix (HLH), NEMO-binding domain (NBD), α-helix (H), coiled coil (CC), and zinc finger (Z).

**Figure 3:**
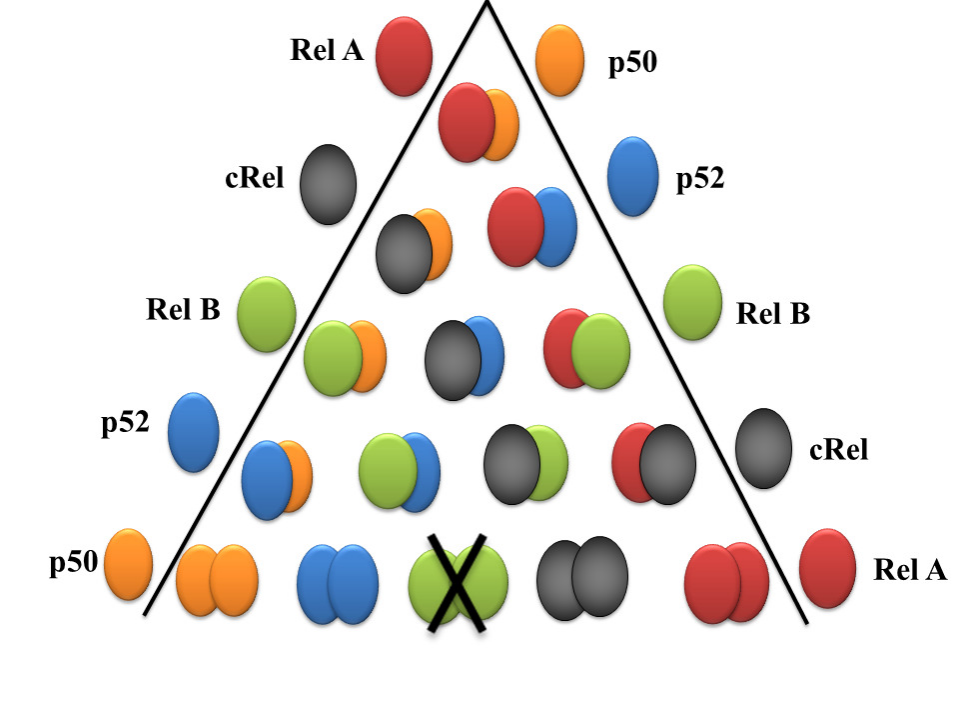
Different stimuli induce specific formation of known homo and hetero NF-κB dimers

The mammalian NF-κB/Rel family possesses five different related monomers (RelA (p65), c-Rel, RelB, NF-κB1 (p50; p105), and NF-κB2 (p52; p100)) that form homo- and hetero-dimers, and bind to 10-base pair kappa B site of promoter region of target genes^20^. The N-terminus of these proteins contains the structurally conserved 300 amino acid sequence called the RHD region, which possesses the dimerization domain (DM), nuclear localization sequence (NLS), DNA-binding domain (DBD) and interaction site with IκBs^21,22^. Three of the family members, RelA, c-Rel, and RelB, have a C-terminal transactivation domain (TAD) that regulates expression of genes. RelA and RelB have two subdomains (TA1/2) of C-terminal transactivation domain^23^. NF-κB1/p105 and NF-κB2/p100 are the inactive precursors of the p50 and p52 proteins, respectively (**Figure 2**)^14^. All monomers of Rel family are capable to form 14 types of homo- or heterodimers and thereby determine the intrinsic NF-κB specificity and its regulation^24-27^, with the exception of RelB, which can only form heterodimers (**Figure 3**).

Different NF-κB dimeric complexes are formed as per cell type and stimulus; some of the physiological important dimers are RelA/p50, cRel/p50 and RelB/p52^22^. RelA and p50 exists in a wide variety of cell types^28^; while c-Rel expression is limited to hematopoietic cells and lymphocytes. The RelB expression is highly specific, being found in the thymus, lymph nodes, and Peyer’s patches^20^. Each NF-κB dimer has the ability to bind with varying affinities to κB sites bearing the consensus sequence GGGRNNYYCC (R, purine: Y, pyrimidine: N, any base) and exhibit their unique functions^29^. However, NF-κB complexes composed only of the family members lacking TAD, such as the p50 homodimers, are known to impose transcriptional repression^30^. For all diverse functions of NF-κB in general, the activity is controlled by a family of regulatory proteins, called inhibitors of NF-κB (IκBs; IκB-α, IκB-β, IκB-ε, IκB-z, Bcl-3 etc)^14,30^ (**Figure 2**).

Three of “typical” IκBs (IκB-α, IκB-β, and IκB-ε), bind to NF-κB proteins and mask their nuclear translocation and DNA binding activity. IκBs also regulates the export of NF-κB proteins from the nucleus, and are thus known for inhibitory processes in multiple ways^31^. Recent investigations suggest that p100, when located in a multimeric complex, may also mediate NF-κB inhibition *in trans*; this activity is termed as IκBδ^32,33^. The complex of IκBs proteins and NF-κB dimers weas originally thought to be retain in the cytoplasm by the NF-κB super repressor IKK. IKK complex is formed by three different subunits: two catalytic subunits IKKα (IKK1 or CHUK), IKKβ (IKK2) and the regulatory subunit IKKg. IKKg is also known as NF-κB essential modulator (NEMO) protein (**Figure 2**). Although IKKα and IKKβ cooperate for IκBs phosphorylation, these proteins differ in the signals that they mediate.

## 3. NF-κB Activation Pathways

There are four models that have been proposed to explain NF-κB activation^34^. NF-κB is activated by numerous pathological and physiological conditions in a very efficient manner. NF-κB also regulates expression of various genes by modulating promoter activity of targets genes^35^.

### 3.1 The IKKβ dependent (classical) pathway

**Figure 4:**
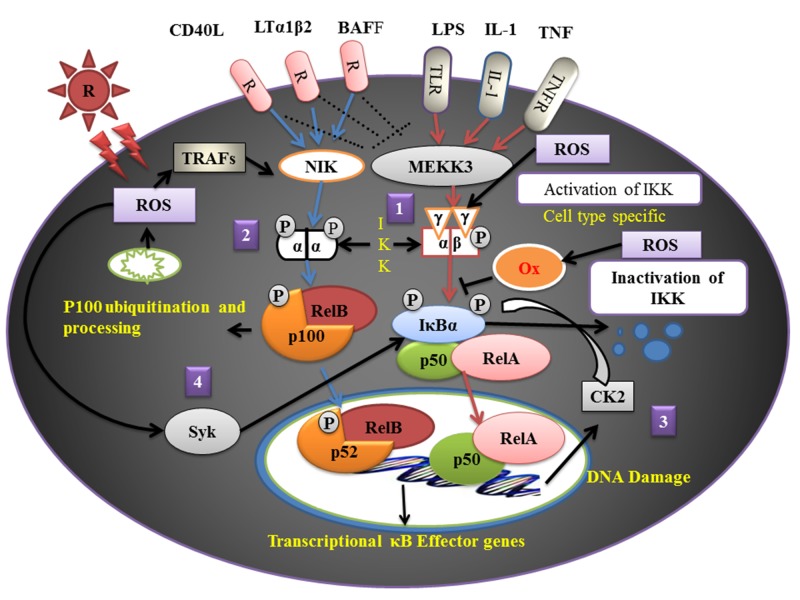
There are four proposed NF-κB signaling pathways in response to various stimuli (1) The canonical pathway (2), the non-canonical pathway (3), atypical pathway and (4) oxidative stress-induced pathway. Downstream binding of the NF-κB proteins to DNA regulates downstream transcriptional of many potential antioxidant, pro-oxidant, cell cycle regulation and anti-apoptotic targets that have been shown in **Supplementary Table 1**.

The IKKβ dependent NF-κB activation has been a well studied signaling event. It is also known as the classical or NEMO (IKK-γ)-dependent or canonical pathway (**Figure 4**). It is induced by several of innate and adaptive immunological agents, and can be turned on within minutes. It principally requires IKKβ components^36,37^. Phosphorylation of IKKβ at Ser177 and Ser181 may occur after stimulation by TNFR, IL-1R, TLR agonists, radiation exposure, TNF-α (tumour necrosis factor-α), PMA (phorbol 12-myristate 13-acetate), interleukins and other factors, which regulate downstream phosphorylation of IκB-α at Ser32 and Ser36, or IκBβ at Ser19 and Ser23, through the function of ubiquitin-dependent protein kinases. Phosphorylated IκB proteins are then ubiquitinated at nearby lysine residues (lysines 21 and 22 of IκBα and lysine 9 of IκB-β), and thus triggers a rapid degradation of IκB proteins by 26S proteasome^38,39^. The rapid degradation of IκB-α, IκB-β, and IκB-ε occurs during classical NF-κB signaling pathway. Phosphorylated p65/p50 (phosphorylation of p65 at Ser536) complex quickly translocates into nucleus and binds to 10-bp kB site or interacts with other transcription factors and regulates expression of various target genes. IκBα is a well known regulatory protein (providing a feedback control) for this pathway. The newly synthesized IκBα enters into the nucleus and prevents NF-κB DNA binding activity and transports NF-κB back into the cytoplasm.

### 3.2 The IKKα dependent (alternative) pathway

Alternative or NEMO-independent or non-canonical pathway is mainly activated during secondary lymphoid organ development, homeostasis and adaptive immunity, and it turns on in few hours^40^. Senftleben et al. first described IKKα dependent pathway in which processing of p100 and activation of p52/RelB is defined as the alternative pathway (**Figure 4**)^39^. In this pathway phosphorylation of IKKα homodimer at Ser176 and Ser180 occurs through the upstream kinase NIK, (NF-κB inducing kinase). This pathway is stimulated by specific TNF receptor family members, such as LTβR, CD40, CD27, CD30, BAFF-R, RANK and others^41^, that signal through the recruitment of TRAF2 and TRAF3. In the resting cells, continuous degradation of NIK prevents non-canonical NF-κB activation^42^.

### 3.3 Atypical pathway

This pathway is essentially independent of IKK and it is mainly triggered in case of UV or chemical-induced DNA damages^43,44^. Evidence suggests that CK2 (formally known as casein kinase II) is a stress-activated protein kinase involved in the transduction of survival signals (**Figure 4**)^45,46^. CK2-mediated IκBα phosphorylation has an important UV-protective function. Jung et al. demonstrated a correlation of ATM with NF-κB in cellular radiosensitivity^47^ and suggested that the loss of ATM function promotes radiosensitivity by activation of NF-κB^47^. Recently, Wu et al.^48^ demonstrated that the cytosolic activation of signaling and sensor complexes (ATM, NEMO, IKK catalytic subunits, and ELKS - an IKK regulatory subunit) are associated with nuclear DNA damage-induced NF-κB activation. This model was proposed on their findings that ATM interacts with NEMO and phosphorylates NEMO at Ser85 after DSBs.

### 3.4 Oxidative stress-induced pathway

Oxidative stress-induced activation of NF-κB signaling is achieved *via* IκB-α tyrosine phosphorylation without degradation of IκB-α by Syk protein tyrosine kinase (**Figure 4**)^49,50^. H_2_O_2_ is one of the central free radical, involved in different cellular processes, including NF-κB activation^51^.The redox-sensitive pathways triggering this activation may vary with everh cell and cell-type^50^. NF-κB is also sensitive to oxidative modifications of Cys62 in p50, which are essential for DNA binding^52,53^. Activation and translocation of NF-κB is stimulated by oxidative circumstances, while its DNA binding affinity is inhibited by the redox sensitive cysteine residue^54,55^. The tyrosine phosphorylation of IκBα by most agents does not lead to IκBα degradation. However, Pervanadate (it is a protein tyrosine phosphatase inhibitor)-induced activation of NF-κB signaling, tyrosine phosphorylation and degradation of IκB-α has been documented^56^. Surprisingly, UV-C induced NF-κB activation is mediated through the degradation of IκB-α, that involves neither phosphorylation of serine nor the tyrosine residue of IκB-α^57^.

## 4. Post translational modifications of NF-κB proteins

**Figure 5:**
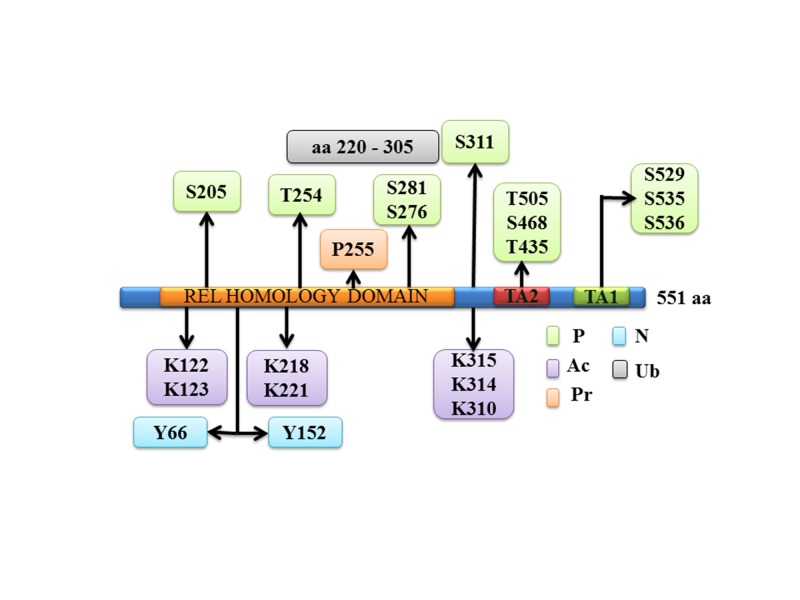
Phosphorylation and acetylation sites within NF-κB p65 Eight Serine three Threonine residues phosphorylation and seven acetylation sites have been identified in the NF-κB p65 subunit. Abbreviations: Ac, acetylation; K, lysine; N, tyrosine nitration; P, phosphorylation; Pr, proline isomerization; S, serine; T, threonine; Ub, ubiquitination; Y, tyrosine.

The mammalian transcription factor NF-κB is activated by over 150 diverse stimuli and thousands of potential NF-κB DNA binding sites have been marked across the genome^13,58^. After degradation of IκBs, activated NF-κB complex moves into nucleus and binds to 10bp defined sequence GGGRNWYYCC (N represents any base, R represents a purine; W represents an adenine or a thymine and Y represents a pyrimidine), which is present in the promoter and enhancer regions of target genes^59^. Moreover, activity and DNA binding affinity of NF-κB transcription factor are spatially and kinetically controlled, thereby regulating expression of its target genes^60^. Within the nuclear compartments, various posttranslational modifications (PTMs) of NF-κB occurs, such as: ubiquitination, acetylation and phosphorylation^61^. Among all NF-κB subunits, most of the post-translational modifications take place in the p65 subunit, which is known to be modified by phosphorylation, acetylation, prolylisomerization, nitrosylation and ubiquitination (**Figure 5** and **Table 1**)^12^. Phosphorylation of p65 unit takes place either in the cytoplasm or in the nucleus, and is mediated by numerous protein kinases. These sites can be modified in a stimulus- and/or cell type-specific fashion by several kinases (**Table 1**)^62-65^.

**Table 1:** The phosphorylation sites of p65, and responsible kinases Acetylation sites of p65 and the corresponding enzymes; * Recently discovered phosphorylation sites

Site	Location	Kinase	Function	Reference
Ser 205*	RHD	unknown	Transcriptional activity	^66^
Ser 276	RHD	PKAc MSK1	Transcriptional activity Captivator binding Transcriptional activity	^67,68^
Ser 281*	RHD	unknown	Transcriptional activity	^66^
Ser 311	RHD	PKCz	Transcriptional activity	^69^
Ser 468	TA2	GSK3β IKKβ IKKα	Transcriptional activity Transcriptional activity Transcriptional activity	^70-72^
Ser 529	TA1	CK II	Transcriptional activity	^73^
Ser 535	TA1	CaMKIV	Transcriptional activity	^74^
Ser 536	TA1	IKKα IKKβ IKKe TBK1 RSK1	Transcriptional activity and stabilization Transcriptional activity and nuclear import Transcriptional activity Nuclear localization Affinity to IκBα	^75-81^
Ser 547*	unknown	ATM-DSB	Transcriptional inhibition of target genes by HDAC recruitment	^82^
Thr 254*	RHD	unknown	Stabilization and Nuclear localization	^83^
Thr 435*	TA2	unknown	Transcriptional activity	^84^
Thr 505	TA2	ATR ChK1	Transcriptional activity	^85,86^
Tyr 66 Tyr 152	RHD	NO treatment	p65 dissociation from p50 and association with IκBα	^87^
				
Site	Location	Enzyme	Function	Reference
Lys 122	RHD	P300, PCAF	Inhibition DNA binding	^88^
Lys 123	RHD	P300, PCAF	Inhibition DNA binding	^88^
Lys 218	RHD	CBP/p300	Unknown	^89^
Lys 221	RHD	CBP/p300	Promoting DNA binding Inhibition IκBα binding	^89^
Lys 310	RHD	CBP/p300	Enhancing transactivation	^89^
Lys 314	RHD	P300	Transcriptional activity	^90^
Lys 215	RHD	P300	Transcriptional activity	^90^

PTMs of p65 can regulate the interaction with co-activators^91^, co-repressors^92^ promoter-bound degradation^93^ and interactions with the basal transcriptional machinery^94^. According to the NF-κB barcode hypothesis the differential modifications of the DNA-binding subunits generate distinct arrays that function through transcription in a highly target gene-specific manner^95^. Other than p65 post-translation modifications, NIK and IKKα (IKK1)-mediated phosphorylation of p105 NF-κB occurs at multiple sites (Ser921, 923, 927, and 932) on its carboxyl-terminus. SCF/β-TrCP-mediated processing of p105 NF-κB produces the 50 kDa active form product, p50^96,97^. NF-κB p50 serine 337 is phosphorylated in response to PKA, which regulates the binding affinity of NF-κB p50 and impacts the NF-κB transcriptional activity. In addition to post-translational modifications, recent studies showed the ability of NF-κB to bind the DNA (NF-κB: DNA) is also regulated by other proteins. A recent report suggests that RPS3 (ribosomal protein subunit 3) interacts with RelA *via *its KH (K Homology) domain and specifically enhances p50: RelA binding affinity with DNA (p50:RelA:DNA)^98^.

## 5. NF-κB regulated proteins and their functions in oxidative stress

Exposure of mammalian cells with low doses of ionizing radiation is known to have variable effects and may generate valuable effects within cells^99,100^. The correct cellular response to ROS following low doses of IR is consequently critical, in order to reduce further oxidative damage, and to maintain cell survival through initiation of cellular signaling, including NFkB pro-survival signaling. Therefore ROS-mediated NF-κB response and thereby regulation of NF-κB target genes may attenuate cell survival. One important way in which NF-κB activity influences ROS levels is by increasing expression of antioxidant and anti-apoptotic proteins. Since NF-κB is known to play a central role in inflammation, some enzymes that promotes the production of ROS are also controlled as well as its targets, particularly in cells of the immune system^31^. A few known or possible NF-κB target genes that may contribute to the protection of cells from ROS-induced cellular damage are mentioned in **Supplementary Table 1^101-191^**.

## 6. Radiation Countermeasures in relation to NF-κB activation

**Figure 6:**
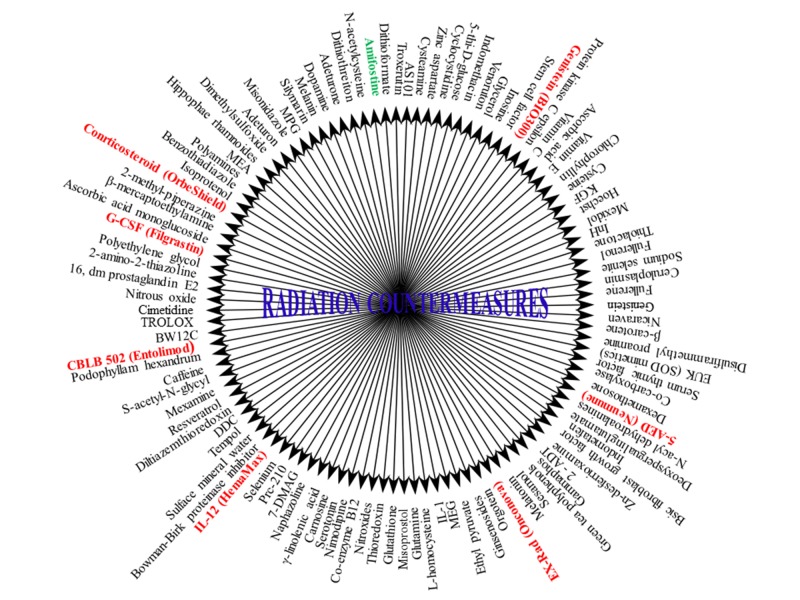
Schematic representation of chemical compounds that have been evaluated for radio-protective potential until today

The radioprotective agent can be described as the “molecule(s) or compound(s) that protects against radiation-induced cellular, tissue injury, when applied before, during, or after irradiation in a specified time period”^192,193^. A number of chemical compounds that are identified and evaluated for radio-protective efficacy may be classified as (Supplementary Table** 2**^194-258^ and **Figure 6**):

- Prophylactic agents,
- Mitigators and
- Therapeutic agents

To date, there are no safe and effective drugs for the protection against ionizing radiation damage. Therefore, a great need exists to identify and develop non-toxic agents that will be useful as radio-protectors or post-irradiation therapies under a variety of operational scenarios. Suppressing of IR-induced cell death or enhancing survival, proliferation, differentiation of cells are the major ways to obtain protection mechanisms against radiation, addressing the massive cell loss in radiosensitive tissues specifically hematopoietic system (HP) and gastrointestinal tract^194-196^. Some of radio-protective agents that are currently in clinical trials are listed in **Supplementary Table 2**. NF-κB plays important roles in immunity and cellular survival in response to radiation exposure and oxidative stress. It is known to reduce programmed cell death or apoptosis by promoting the expression of anti-apoptotic proteins and antioxidant molecules associated with enhanced radio-resistance, whereas its deletion in mice results in hypersensitivity to ionizing radiation-induced GI damage^21,197^.

NF-κB also regulates the production of a wide variety of cytokines in a cell type specific manner. Some of these cytokines induce proliferation and survival of hematopoietic stem cells, thereby promoting bone marrow recovery and tissues regeneration following irradiation. Therefore, pharmacological activation of NF-κB may be considered as a possible approach for radioprotection / mitigation. In this review, we have discussed some radioprotectors/ mitigators, specifically in relation to their efficacy for activation of NF-κB. Great efforts have been directed towards recognizing the role of TLRs (Toll Like Receptor)-mediated responses to microbes (viruses, bacteria, fungi) for the development of novel therapies in autoimmune allergic diseases, malignancy and other infections^259^. Investigations of TLR agonists are one of the global recent interests, for use in the preparation of immune-modulators. TLR agonists include: small molecules, pathogen derived DNA, RNA, proteins, lipids, which target one or more of the toll-like receptors, including TLR 2-9. Bacterial flagellin, the natural ligand of TLR5, was found to have radioprotective effects in rodents and nonhuman primates^260^. Recently, Cleveland Bio-Lab has developed the new pharmacological CBLB series, including CBLB502, for radiological emergencies. CBLB502 is a rationally designed derivative of Salmonella flagellin. It is substantially less immunogenic than full length flagellin and possesses its TLR5-dependent NF-κB–inducing activity and radioprotective ability^208^. Moreover, CBLB502 protected mice from dermatitis and mucositis associated with local fraction irradiation of head and neck area modelling radiation treatment of patients with head and neck cancer and also was shown to be effective as a tissue protectant in mouse models of renal ischemia-reperfusion injury^261^. A single dose of CBLB502 (0.2mg/kg body weight) 30 min prior to 13 Gy of TBI to NIH-Swiss mouse offered 87% protection. Administration of CBLB502 even up to 1 h post-irradiation results in greater than 90% survival after 9 Gy. CBLB502 also showed radio-protective efficacy in lethally irradiated rhesus monkeys^208^. Burdelya et al, recently showed that liver was the primary responsive organ for CBLB502 and CBLB502-mediated radioprotection of the HP system. The radioprotection occurred by factors secreted by responsive liver hepatocytes. A strong suppression of growth of tumor cells in the liver, regardless of their TLR5 status, was also observed^209^.

Recently, a lipopeptide of Mycoplasma arginini has been reported to act as a TLR 2/6 agonist. This novel radiation countermeasure, CBLB 613, has been observed as possible radio-mitigator for humans against radiation induced lethality^262^. CBLB613 significantly protected mice against a lethal dose of γ-radiation with no observable toxicity at 1.79 mg/kg body weight and 1 mg/kg body weight for single and repeated doses, respectively. In irradiated CD2F1 mice it stimulates bone marrow cellularity, enhances production of cytokines, such as interleukin-1β (IL-1β), IL-6, IL-10, IL-12, keratinocyte-derived chemokine, granulocyte colony-stimulating factor (G-CSF), granulocyte-macrophage colony-stimulating factor (GM-CSF), and tumour necrosis factor-1α (TNF- α), and reduces radiation-mediated cytopenia. CBLB613 exhibits substantial dose reduction factor of 1.25.

The baicalein is a bioactive flavonoid, which has been shown to have antioxidant, anti-inflammatory and anti-hepatotoxic properties in both *in vitro* and *in vivo *conditions^263,264^. Treatment with baicalein inhibits the inflammatory signaling pathways involving ERK (extracellular signal-regulated kinase), Akt and nuclear factor-κB (NF-κB) activities in vascular smooth muscle cells^265^. A recent study showed that γ-irradiation with baicalein reduces lipid and protein oxidation in rat liver. Damaging effects of IR are generally mediated through the production of reactive species (RS), and a substantial increase in RS levels induces cellular damage and decrease in antioxidant enzymes, as well as activates intracellular signaling pathways that activate the expression of many inflammatory genes. The IR induced molecular responses may also be characterized as increased cyclooxygenase-2 (COX-2) level, inducible nitric oxide synthase (iNOS) and vascular adhesion molecule-1 (VCAM-1) expressions that also initiates the activation of the transcription factor NF-κB^266^. The key role played by NF-κB activation in the process of inflammation has been reported to be closely associated with a redox-sensitive signal cascade that includes MAPKs (ERK, c-Jun N-terminal kinase (JNK] and p38) and Akt^266,267^. However, activation of the Akt signaling pathway has been known to reduce forkhead box-O (FOXO) transcription activity^268^, and is involved in cytoprotective effects against oxidative stress^269^. Irradiation of mice showed an enhancement of NF-κB-mediated inflammatory factors due to the oxidative damage, and the inactivation of FOXO and its target genes, such as catalase and SOD. However, baicalein (5mg/kg bw/day) has the ability to suppress radiation-induced inflammatory consequences, by down regulating NF-κB and up-regulating FOXO activation^270^. Furthermore, baicalein inhibited radiation induced phosphorylation of MAPKs and Akt, which are upstream kinases of NF-κB and FOXOs. These observations also suggest that baicalein has a radioprotective effect against NF-κB mediated inflammatory response, through MAPKs and the Akt pathway, which is complemented by the protective effects on FOXO and its target genes, such as catalase and SOD.

DNA double-strand breaks (DSBs) are the most deleterious form of DNA damage and numerous *in vitro* studies have analyzed the DSB repair system that is activated after exposure to ionizing radiation. DSBs rapidly trigger the activation of NF-κB pathway *via* NEMO^48,271^. The death-domain protein PIDD was originally identified as an early p53-inducible gene and is implicated in p53-induced apoptosis^148^. PIDD is a mediator of the DNA-damage-activated stress response and is involved in genotoxic stress-induced NF-κB activation^271,272^. PIDD expression enhances genotoxic-stress-induced NF-κB activation through augmented sumoylation and ubiquitination of NEMO^272^. Corilagin (ß-1-O-galloyl-3, 6-(R)-hexahydroxydiphenoyl- D-glucose) is a member of the tannin family and has been isolated from medicinal plants, such as the *Phyllanthus* sps^273^. Corilagin has antioxidative, atherogenic, and hypertensive effects in various models^273-276^. A preliminary *in vitro* study suggested that corilagin has anti-inflammatory activity^277^. The activation of microglia and release of pro-inflammatory cytokines post irradiation are regarded as the key effectors of RIBI. Recent, studies demonstrated that corilagin exhibited anti-inflammatory activity in irradiated B7-2 cells by suppressing the release of pro-inflammatory cytokines and mediators. Corilagin suppresses the transcription of pro-inflammatory cytokine genes, through effects on the DSB-triggered NF-κB signaling pathway^278^.

Ex-RAD employs a novel mode of action, involving the enhancement of internal DNA repair pathways, which significantly reduces the levels of p53, p21, bax, c-abl and p73 proteins-key players in the DNA damage cascade induced upon exposure to 8.0 Gy gamma irradiation^207^. These mechanisms can cause a halt in cell death pathways and lead to increased recovery and survival of irradiated cells. These novel mechanisms of action attended by minimal side effects suggest that Ex-RAD could be useful both as a prophylactic and mitigative agent. Ex-RAD (4-carboxystyryl-4-chlorobenzylsulfone, sodium salt; or ON 01210.Na] is a synthetic small-molecule radioprotective compound (from Onconova Therapeutics, Inc. (OTI)) that is active in male C3H mice^207^ when administered 24 h and 15 min (two injections) before total body irradiation (TBI). Although Ex-RAD had been shown to be an inhibitor of apoptosis *in vitro*, it is not recognized whether a parallel mechanism is occurring *in vivo*^207^. In numerous cell-based and complete animal models, Ex-RAD has revealed to have potential for defense from radiation injury when administered either before or after radiation exposure. The drug is currently in Phase I clinical trials in humans. In decision, Ex-RAD usage mitigates potentially life-threatening neutropenia and bone marrow overthrow and, in turn, stimulates bone marrow retrieval, decreases radiation induced phosphorylation of p53 signaling, and enhances survival of acutely irradiated mice^279^. In addition to mitigating of hematopoietic damage, Ex-RAD also moderates intestinal injury. However, the molecular mechanisms elaborated in Ex-RAD’s promotion of recovery of hematopoietic and GI tissues warrant further study.

## 7. Conclusion

Radiation-induced injuries and lethality are well described at clinical level and understanding of mechanisms of tissue responses in the event of radiation exposure has gained much attention in recent years. The quest to search a potent radiation countermeasure which can ameliorate radiation syndrome and at the time exhibits no toxicity for human consumption is prevalent since past decades. However, even after the existence of a lot of literature available on radiation counter-measures only handful of identified drugs seem promising for human use. Based on prudent dissection of complicated series of signaling changes within multiple pathways, it might be possible to rationally combine inhibitors of these cascades, to repair damaged bio-molecules, activation of intracellular pathways, stress receptor activation, to achieve radiation protection. As a stress sensor, NF-κB is a crucial component of the cell’s protective response to radiation and therefore an attractive target in the new therapeutic lines to fight cancer or radiological emergencies. NF-κB is now documented as an important player in several critical steps for development of radiation countermeasures. **

Recently, focus of radiation protection has shifted to test the radioprotective potential of plant products and herbs in the hope that one day it will be possible to find a suitable pharmacological agent that could protect humans against the deleterious effects of ionizing radiation in clinical and other conditions as well as during nuclear terror attack. Majority of plants and herbs described in this review have medicinal properties and are being used in traditional Ayurvedic or Chinese systems of medicine to treat various ailments in humans. Our review provides a broad idea on the physicochemical role of ionizing radiation on cellular systems and highlights the importance of developing new natural radioprotectants. Medicinal plants like *Aconitum heterophyllum, Bergenia stracheyi, Bunium persicum, Dactylorhiza hatgirea, Ephedra gerardiana, Pichorrhiza kurroa*, etc., are some of the plants that need elaborate investigations. Furthermore, some radioprotectants may boost their own efficacy in combination therapies Fractionation guided evaluation may result in the development of ideal radioprotectors in the near future.
