# Supplementary material for: NF-kB as a key player in regulation of cellular radiation responses and identification of radiation countermeasures
Source: Discoveries (Craiova). 2015 Mar 31;3(1):e35. doi: 10.15190/d.2015.27 (PMC7159829; doi:10.15190/d.2015.27)
Supplement: Supplementary file 1 [file discoveries-03-035-s001.pdf]

Discoveries Journals
publishing the future

DISCOVERIES 2015, 3(1): e35
DOI: 10.15190/d.2015.27

Supplementary Information

NF-κB as a key player in regulation of cellular radiation responses and identification of radiation countermeasures

Vijay Singh, Damodar Gupta, Rajesh Arora*

Division of Radiation Biosciences, Institute of Nuclear Medicine & Allied Sciences, Brig SK Mazumdar Marg, Timarpur, Delhi, India.

**Corresponding author*: Damodar Gupta, PhD, Division of Radiation Biosciences, Institute of Nuclear Medicine & Allied Sciences, Defence Research and Development Organisation, Brig. SK Mazumdar Marg, Timarpur, Delhi 110054, India; Phone: 011-23905370; Fax: 011-22919509; Email: damodar@inmas.drdo.in

Submitted: January 15, 2015; *Revised:* March 17, 2015; *Accepted:* March 19, 2015; *Published:* March 31, 2015;
Citation: Vijay Singh, Damodar Gupta*, Rajesh Arora. NF-κB as a key player in regulation of cellular radiation responses and identification of radiation countermeasures. *Discoveries* 2015, Jan-Mar; 3(1): e35. DOI: 10.15190/d.2015.27

SUMMARY OF SUPPLEMENTARY INFORMATION:

► **Supplementary Table 1**. ***The list of NF-kB regulated proteins in response to radiation and countermeasure agents***

► **Supplementary Table 2. *The list of radiation countermeasure agents based on NF-κB pro-survival signaling***

Supplementary Table 1. ***The list of NF-kB regulated proteins in response to radiation and countermeasure agents***

Antioxidant and NF-κB targets			
**Enzyme**	**Regulation**	**Activity of the enzyme**	**References**
MnSOD	It is also known as SOD2 and top most well-known NF-κB target. MnSOD deficient mice die prenatally after birth due to massive oxidative stress. It is down-regulated in many diseases, and may be up-regulated in some cancers.	MnSOD is a mitochondrial enzyme that protects cells from oxidative stress by converting $\cdot O^-_2$ into H_2O_2.	101-104
Cu, Zn-SOD	It is also known as SOD1. Mice lacking SOD2 have a shortened life span and may develop hepatocellular carcinoma due to insistent oxidative damage.	Cytoplasmic enzyme, catalyzes the dismutation of $O_2^{-\cdot}$ into H_2O_2.	105
Ferritin Heavy Chain (FHC)	FHC is the second-most well-known NF-κB target that protects from oxidative damage	FHC is an iron storage protein, which does not directly scavenge ROS, but protects the cell from oxidative damage by preventing iron-mediated generation of highly reactive $^\cdot OH$ radicals from H_2O_2 (Fenton reaction). FHC may synergizes with MnSOD, promote the breakdown of H_2O_2 into water by peroxidases and catalases.	106, 107
Catalase	Little evidence exists for the regulation of Catalase by NF-κB. One report suggests that Catalase promoter is bound by p50 homodimers and down-regulated when canonical NF-κB activation occurs.	Catalase is a common enzyme found in nearly all living organisms exposed to oxygen (such as vegetables, fruit or animals). It catalyzes the decomposition of H_2O_2 to H_2O and $O_2^{\cdot}$	108, 109
Thioredoxin-1 (Trx1)	Trx1 is most important cellular antioxidant regulated by NF-κB. Inactivation of Trx1 in mice results in early embryonic lethality.	Thioredoxin-1 is expressed in the cytoplasm and nucleus. Thioredoxins protect cells/bio-molecules from oxidative stress by means of their 2-cysteine active site that reacts with ROS and is also able to reduce oxidized proteins. They also serve as hydrogen donors to the thioredoxin-dependent peroxide reductase.	110, 111
Thioredoxin-2 (Trx2)	Trx2 is regulated by NF-κB. It is localized within the mitochondria and is also crucial for cell survival. Deletion of the Trx2 causes massive apoptosis due to the damage accumulation by intracellular ROS, resulting in early embryonic lethality in homozygous mice.	Thioredoxin-2 is localized within the mitochondria and is crucial for cell survival. It reacts with ROS and reduces oxidised proteins.	112, 113
GST-pi	NF-κB up-regulates the expression of GST-pi in oxidative stress and play important role in the repair of damage caused by increased oxidative stress.	GST-pi is a phase II enzyme that catalyzes the reaction of the GSH thiolate to toxic electrophilic compounds, thus allow highly reactive	114-117

	Distraction of the gene encoding GST-pi protects HCT116 cells from oxidative stress and resultant apoptosis under growth-limiting conditions.	carcinogens or radicals to be eliminated by excretion machinery	
Metallothionein 3 (MT3)	MT3 has been shown to be an NF-κB target in keratinocytes and fibroblasts.	Metallothioneins are low-molecular-weight, cysteine-rich proteins, which bind to many different metals. In addition of reducing toxicity induced by metal, the cysteine residues in metallothioneins can scavenge $O_2^{-\cdot}$ and $^{\cdot}OH$ radicals.	118-120
NQO1	NF-κB regulates expression of NQO1 in response to the DNA cross-linking induced by mitomycin C. NQO1 deletion prevents the activation of NF-κB.	This FAD-binding protein is a cytoplasmic 2-electron reductase that reduces quinones to hydroquinones. Since it is a 2-electron reductase, its enzymatic activity prevents the one electron reduction of quinones that produces radical species.	121-123
Heme oxygemase (HO-1)	HO-1 is up-regulated by NF-κB and other transcription factors, in response to oxidative stress and hypoxia.	HO-1 catalyzes heme into carbon monoxide and biliverdin, which is then reduced to bilirubin by biliverdin reductase. Bilirubin is a potent antioxidant, it is thought that HO-1 is therefore protective from oxidative stress	124, 125
Glutathione peroxidase-1 (Gpx1)	In response to oxidative stress glutathione peroxidase is upregulated by NF-κB in skeletal muscles cells. In addition, all five of NF-κB subunits in U937 are bound to the glutathione peroxidase promoter.	Gpx1, is an antioxidant enzyme, abundantly found in cytoplasm that catalyzes the H_2O_2 into water using glutathione as a substrate. It also can diminish lipid peroxides, as well as peroxynitrite.	108, 126, 127
Dihydrodiol dehydrogenase (DDH1; AKR1C1)	DDH1 is one of the many dehydrogenase enzymes regulated by NF-κB . It contains putative binding sites for liver specific factors including NF-IL6 and HNF-5 sites and AP-1, AP-2 and NF kappa B-like sites.	DDH1 is a phase-2 aldoketo reductase and oxidizes transdihydrodiols of polycyclic aromatic hydrocarbons. Like many phase-2 enzymes that activate toxic compounds to eliminate them from the body, the reactive products downstream of its reaction have been associated with induction of ROSEctopic expression of DDH1 has been shown to lower the basal levelsof ROS in some cell types, suggesting that DDH1 can act as a protective enzyme.	128, 129
**Pro-ROS NF-κB targets**			
**Enzyme**	**Regulation**	**Activity of the enzyme**	**References**
Gp91 phox	During the inflammatory process, expression of the phagocytic *NADPH oxidase NOX2 (gp91 phox)* is dependent on NFκB, and also induced by NFκB.	NADPH oxidase enzymes are specifically dedicated to the production of ROS, which plays important role in immune defenses, and also initiates various cell signaling.	130-132
Xanthine Oxidase/ Dehydrogenase (XOR)	XOR, or Xanthine Oxidoreductase is regulated by NF-κB.	XOR; enzyme that exists in two forms that catalyze either reduction or oxidation reactions. The dehydrogenase form is the most dominant form *in vivo*, however it may be converted into the oxidase form through the oxidation of its protein	133, 134

		sulphydryl groups.	
Nitric oxide synthases (iNOS/NOS-2 and nNOS/NOS-1	The *iNOS (NOS2)* is greatly up-regulated by NF-κB in vascular smooth muscle cells and macrophases. The expression of nitric oxide synthases can potentiate ROS damage as well as signaling. *Neuronal Nitric Oxide Synthase* or *nNOS (NOS1)* is also an NFκB target in human neuroblastoma cells.	Technically NOS actually produces nitrogen species (i.e., nitric oxide, or NO) and not the reactive oxygen species. NO reacts with superoxide leading to formation of the highly reactive peroxynitrite. While peroxynitrite itself is highly reactive as both an oxidant and nitrating agent, it also reacts with CO_2 to form Nitrosoperoxycarbonate ($ONOOCO_2$-), which then homolyzes to form carbonate (CO_3·-) and nitrogen dioxide radicals ($NO_2^·$). Peroxynitrite may cause various kinds of cellular damage, including damage to DNA, and can activate cell death pathways. Thus the expression of nitric oxide synthases can potentiate ROS damage as well as in the signaling.	135-139
Cyclooxygenase-2 (COX-2)	COX-2 involved in inflammation and is a known NF-κB target.	COX-2, also known as Prostaglandin G/H synthase 2 is involved in inflammation that converts arachidonic acid into prostaglandin H2 (PGH_2) by a free radical mechanism involving a protein tyrosyl radical generated by cooperation from a heme prosthethic group. During the second step of the reaction that produces PGH_2, superoxide is also generated. Thus, superoxide is a side product of this reaction, and may contribute to oxidative stress as well as in the signaling.	140
LOX-5 and LOX-12	*LOX-5* and LOX-*12* that involved in arachidonic acid metabolism and generation of ROS have also been reported to be NF-κB targets.	In addition, the metabolic products of LOX-12 and LOX-5, 12(S) - hydroxyeicosa tetranoic acid and leukotriene B4, respectively, have been shown to activate and induce NADPH oxidases.	141-143
Cytochrome p450 (Cyp p450)	Cyp p450, phase I enzymes that detoxify toxic compounds, and is regulated by NF-κB.	Cytochrome p450 enzymes, have long been known to produce ROS when uncoupled, particularly H_2O_2 and hydroxyl radicals.	144, 145
Cyp2E1, Cyp7b and Cyp2C11	*Cyp2E1, Cyp2C11, and Cyp7b* are recognized NF-κB targets. Cyp2E1 and Cyp2C11 are both down-regulated by NF-κB, while Cyp7b is up-regulated.	Both Cyp2E1 and Cyp2C11 are known to be able to produce ROS through uncoupled reactions	146-149

Cell cycle regulation & Anti-apoptotic proteins

**Enzyme**	**Regulation**	**Activity of the enzyme**	**References**
Inhibitor of apoptosis proteins 1/2 (c-IAP-1/2)	The TNF-induced NF-κB activity induces transcription of a set of genes codes for anti-apoptotic proteins, such as c-IAP-1 and c-IAP-2, which can block caspase functions.	The IAP family consists of an evolutionarily conserved group of apoptosis inhibitors containing a conserved 70 amino acid BIR domain. Human members of the family include c-IAP1, c-IAP2, XIAP, Survivin, Livin and	150-153

		NAIP. In general, the IAP proteins function through direct interactions to inhibit the activity of several caspases, including caspase-3, caspase-7 and caspase-9. In addition, binding of IAP family members to the mitochondrial proteins Smac, blocks its interaction with caspase-9, thereby allowsthe processing and activation of caspase.	
XIAP	Tang et al. showed the NF-κB induced XIAP, negatively modulates TNFα mediated JNK activation	XIAP directly inhibits key activated caspases, in both pathways. XIAP binds to the effecter caspase-3 and caspase-9, *via* its BIR domain, hindering substrate engagement by the proteases and leading to the resistance of FAS-mediated apoptosis.	154
c-FLIP	c-FLIP, an anti-apoptotic factor is regulated by NF-κB in response to calpain 3, the protein involved in limb-girdle muscular dystrophy type 2A. One other study suggest that, the c-FLIP controls NFκB activation and life/death decisions in lymphocytes and DCs. together, Recently, another study reveals a novel link between NF-κB and PI3K/Akt and establishes c-FLIP as an important regulator of FasL-mediated cell death.	Cellular FLIP (FLICE inhibitory proteins) is a regulator of apoptosis. However, at physiological levels it is thought that the binding of FLIP to the DED of FADD results in inhibition of caspase-8 processing.	155- 157
Bcl-2	Bcl-2 gene is transcriptionally regulated by NF-κB in response to TNF-α in prostate cancer. The translocation of NF-κB correlates with bcl-2 and bax expression and that the NF-κB/bcl-2 pathway may be associated with a poor response to neo-adjuvant doxorubicin based chemotherapy.	Bcl-2 exerts a survival function in response to a wide range of apoptotic stimuli through inhibition of mitochondrial cytochrome c release. It has been implicated in modulation of mitochondrial calcium homeostasis and proton flux. Mutation of Bcl-2 at Thr56 or Ser87 inhibits its anti-apoptotic activity during glucocorticoid-induced apoptosis of T lymphocytes. Interleukin 3 and JNK-induced Bcl-2 phosphorylation at Ser70 may be required for its enhanced anti-apoptotic functions.	158-162
Bcl-xL	NF-κB directly regulates the expression of distinct pro-survival factors in the Bcl-2 family, such as Bcl-xL and Bcl-1/A1, in human HT1080-hc-rel and HeLa-derived HtTA-CCR43 cell lines.	Bcl-xL prevents apoptosis through two different mechanisms; by hetero-dimerization with an apoptotic protein to inhibit its apoptotic effects and by its direct pore-forming effect on the outer membrane of mitochondria, which helps to maintain a normal membrane state under stress conditions. Bcl-xL is phosphorylated by JNK following treatment with microtubule-damaging agents such as paclitaxel, vinblastine and nocodazole.	163-168
Cyclin D1	IR-induced NF-κB activation could be involved in cell cycle arrest and prevention of apoptosis to allow cells	The activity of CDK4 and CDK6 is regulated by T-loop phosphorylation by the abundance of their cyclin partners (the	169-175

	to repair damaged DNA and NF-κB is able to regulate the cell cycle via induction of G1/S protein cyclin D1.	D-type cyclins) and by association with CDK inhibitors of the Cip/Kip or INK family of proteins. The inactive ternary complex of cyclin D/CDK4 and p27 Kip1 requires extracellular mitogenic stimuli for the release and degradation of p27 concomitant with a rise in cyclin D levels to effect progression through the restriction point and pRb-dependent entry into S-phase. The active complex of cyclin D/CDK4 targets the Rb protein for phosphorylation allowing the release of E2F transcription factors that activate G1/S-phase gene expression. Levels of cyclin D protein drop upon withdrawal of growth factor through down regulation of its protein expression and through phosphorylation-dependent degradation.	
Cyclin B1	The accretion of cyclin B1 and 14-3-3ζ immunoreactive proteins can be induced by low-dose ionizing radiation in mouse skin epithelial JB6P+. Inhibition of NF-κB, reduced the expression of cyclin B1 and 14-3-3ζ and diminished LDIR-induced adaptive resistance.	The entry of eukaryotic cells into mitosis is regulated by activation of cdc2/cdk1 at the G2/M transition. This activation is a multi-step process that begins with the binding of the regulatory subunit, cyclin B1 to cdc2/cdk1 to form the mitosis-promoting factor (MPF). MPF remains in the inactive state until phosphorylation of cdc2/cdk1 at Thr161 by cdk activating kinase (CAK) and de-phosphorylation od cdc2/cdk1 at Tyr15 by cdc25C. Five cyclin B1 phosphorylation sites (Ser116, 126, 128,133 and 147) are located in the cytoplasmic retention signal domain and are thought to regulate the translocation of cyclin B1 to the nucleus accumulation and initiation of mitosis.	176-179
GADD45β	GADD45β acts as an essential factor in the balance of anti-versus pro-apoptosis due to radiation-induced cytokine activation. Recent progress demonstrated that overexpression of GADD45β blocks TNF-α induced activation of JNK. It is shown that antisense blocking of GADD45β inhibits NF-κB and ERK activity.	Growth arrest and DNA-damage-inducible, beta, also known as GADD45B, is a protein which in humans is encoded by the GADD45B gene. GADD45 α, β and γ are an evolutionarily conserved, homologous family of nuclear proteins that function as stress sensors for cellular physiological and environmental damage. GADD45 proteins are required for the activation of the G2/M checkpoint induced by UV radiation or alkylating agents. GADD45-induced G2/M checkpoint is regulated through inactivation of cdc2-cyclinB1 kinase. GADD45 forms a complex with p21 and Cdc2 and may serve as core for interaction with other cell cycle regulators.	180-185
Ku70/80	The expression of Ku70 and Ku80 are mediated by NF-κB and Cyclooxygenase-2 in human gastric	Ku is a heterodimeric protein composed of two subunits (Ku70 and Ku80) originally identified as autoantigens	186-189

	cancer cells.	associated with several autoimmune diseases including scleroderma, polymyositis and systemic lupus erythematosus. The Ku70/Ku80 heterodimer has ATP-dependent DNA helicase activity and functions as the DNA-binding regulatory component of DNA-dependent protein kinase (DNA-PK). DNA-PK has been shown to phosphorylate many proteins, including p53, serum response factor, c-Jun, c-Fos, c-Myc, Oct-1, Sp-1 and RNA polymerase II. The combined activities of Ku70/Ku80 and DNA-PK implicate Ku in many cellular functions, including cell-cycle regulation, DNA replication and repair, telomere maintenance, recombination and transcriptional activation.	
HER-2	Recently, it observed that NF-κB may activate HER-2 expression. The 10-bp NF-κB DNA binding sequence, i.e., consensus site (GGGACGACCC; located between -364 and 355), is found in the promoter region of HER-2.	The HER2 (ErbB2) proto-oncogene encodes a 185 kDa transmembrane, receptor-like glycoprotein with intrinsic tyrosine kinase activity. HER2 is a key therapeutic target in the treatment of breast cancer and other carcinomas and targeting the regulation of HER2 degradation by the c-Cbl-regulated proteolytic pathway is one potential therapeutic strategy.	190, 191

Supplementary Table 2. ***The list of radiation countermeasure agents based on NF-κB pro-survival signaling***

Compound	Protective Efficacy	Probable mechanism of action	References
Amifostine (WR-2721)	Amifostine (previously known as WR-2721) has high degree of protection with a radiation dose modifying factor 2.7 when given to mice IP 30 minutes before exposure to gamma radiation	Free radical scavenging and hydrogen donation	198-201
Prc-210	It is a bifuctional aminothiol antioxidant, protected from TBI (12Gy) lethality when injected IP 30 minutes before irradiation. It also decreased IR-induced mucositis when applied topically for 30 minutes before irradiation (20Gy).	Potential as topical or systemic radioprotector, free radical scavenging, antioxidant	202
Selenium	Selenium is protective when administered (1.6mg/kg WB, orally or paritonially) 24 hours before exposure to 9 Gy radiation.	Free radical scavenging,	203, 204
Ex-Rad	Ex-Rad showed radioprotective efficacy if applied 14 hour and 15 minutes before 8Gy radiation exposure. It does not protect mouse prostate cancer cells from RT (10Gy) but potentiate RT effectiveness *in vivo*	Potential as radioprotector	205-207
CBLB502	Rationally designed CBLB502 (from flagellin) rescued > 87% of mice when given as a single SC injection 30 min before 13Gy TBI and also increased survival when administered 1h after 9 Gy TBI. Importantly, CBLB502 was also found to be effective as a radioprotectant in rhesus monkeys subjected to 6.5Gy TBI.	Protect both hematopoietic and GI components of ARS without causing systemic inflammation (TLR 5 agonist)	208, 209
R-Pam2C-SKKKK (CBLB601)	Lipopeptides stimulates NF-kB via TLR2/6 pathways. CBLB601 was evaluated as radioprotector in mice. It protected hematopoietic system and shown to prolong survival, when administered IP or IM, 24 hour before followed 10Gy TBI.	Protect hematopoietic system (TLR 2,6 agonist)	210
R-Pam2C-VQGEESNDK (CBLB612)	CBLB612 showed radioprotective activities if applied 24 hour before irradiation as well as mitigation applied 1 hour after irradiation.	Radioprotector and mitigator (TLR 2,6 agonist)	210, 211
R-Pam2-CGETDK (CBLB613)	Increase survival when injected 6h after 9.6Gy TBI. Although these compounds are powerful inducers and mobilizers of BM stem cells.	Induce cytokines production	212
5-AED	5-AED has ability to reduce mortality, thrombocytopenia, and neutropenia in irradiated mice and nonhuman primates.	Protect hematopoietic progenitor cells *in vitro* / *in vivo*	213-215
17-DMAG	Geldanamycin derivative 17-DMAG protects mice and improves mice survival against lethal dose of gamma radiation. It reduces the radiation induced activation of iNOS synthase pathway, thereby blocking apoptosis and autophagy. This drug also inhibited the radiation induced activation of p53-bax signal transduction.	Anti-apoptotic and autophagy and inhibit iNOS pathway.	216, 217
Diethyldithiocarbamate (DDTC)	DDTC has antioxidant and radioprotective abilities. DDTC protected rat liver microsomal	Radical scavenging potential	218, 219

	membranes *in vitro* from peroxidative damage in lipids resulting from 50Gy gamma-radiation. It also protected plasmid pBR322 DNA from radiation-induced strand breaks. An oral administration of DDTC to mice before whole body gamma-radiation exposure (4 Gy) resulted in a reduction of radiation-induced lipid peroxides.		
Interleukin 1 and FGF 1/2	IL-1 protected irradiated mice when given either 20hours before or 2hours after irradiation. Radioprotection with a DRF in the range of 1.15 to 1.25 was maximized when given 20hours before radiation at doses of 4 or 8µg/kg body weight, respectively. Acidic fibroblast growth factor (FGF) 1 showed DRF of 1.16.	Protect hematopoietic system and Tissue regeneration	220-222
TNF-α	TNF-α showed radioprotective efficacy in mice. It has been reported that TNF-α does not protect tumor cells from radiation but protects only normal cells. On the other hand it is also reported that specific inhibition of TNF-α receptor by genetic knock-out protected lungs from radiation.	Protect normal cells specifically and also found to protect lungs from radiation induced damages	223, 224
Podophyllam hexandrum (Royle)	*Podophyllum hexandrum* has been shown to reduce radiation injuries and especially the haematopoietic syndrome in adult mice. It enhanced survival of mice and increased levels of liver GST and SOD besides intestinal SOD. It also prevents radiation-induced neuronal damage in postnatal rats exposed *in utero* and mitochondrial system.	Protect haematopoietic and neuronal damage, Mitochondria free radical scavenging	225-230
Hippophae rhamnoides (Linn.)	*Hippophae rhamnoides* (Sea Buckthorn) has antioxidant, anti-inflammatory, anti-microbial and immunostimulatory potential. Its aqueous extract enhances the survival of strain 'A' mice when given 30 minutes before whole body γ-irradiation. It provides protection to the gastro-intestinal system against lethal TBI. Administration of a hydroethanol (50:50 v/v) extract, 30 minutes before irradiation increased the number of surviving crypts in the jejunum by a factor of 2.02 and villi cellularity by 2.5 fold.	Protect gastro-intestinal system, Mitochondria, free radical scavenging	231- 233
Sesamol	Significantly reduced γ-radiation-induced micronuclei and dicentrics in human lymphocytes	Protect GI tract, free radical scavenging	234
Troxerutin	During radiotherapy of head and neck cancer, administration of a mixture of troxerutin and coumarin offered protection to salivary gland and mucosa. It also inhibited lipid peroxidation in membranes of sub-cellular organelles as well as normal tissues of tumor-bearing mice exposed to γ-radiation. And differential protection of DNA in blood leukocytes and bone-marrow cells and not in cells of tumor in whole body irradiated tumor bearing mice	Protect DNA, free radical scavenging	235, 236
Ethyl pyruvate	Ethyl pyruvate has ROS scavenging and anti-inflammatory potential. It enhances the resistance of mouse hematopoietic system against 10Gy of	Protect hematopoietic injury and may be used for RT	237-239

	radiation exposure *in vitro* and significantly increased mice survival exposed to 9.75Gy TBI. Nevertheless it was recently shown to inhibit the growth of hepatic tumor *in vivo*		
G-CSF (Filgrastim)	Over the past decades, preclinical study suggested that cytokines can facilitate recovery from ARS by promoting cell survival and proliferation. However, clinically only G-CSF and GM-CSF are recommended for ameliorating IR-induced myelosuppression. It helps to recovery of the bone marrow and induced growth factors that stimulate the blood cells to multiply. G-CSF has high potential and well-documented therapeutic effects and may receive full licensing approval by the US FDA in the future.	Myelosuppression, activation of immune system	240- 243
Genistein (BIO-300)	It protects mice, when injected 24h (SC) or 4 days (orally) before or after 9.5Gy whole body radiation exposure. Moreover, it promote the recovery of myeloid and erythroid lineages and reduced lung injury. It also increased the sensitivity of tumor	Potential as radio-protector and radio-mitigator Myeloid cells protection	244-247
Melatonin	Melatonin, (N-acetyl-5-methoxytryptamine), is a well-known antioxidant that protects DNA, lipids, and proteins from free-radical induced damage. melatonin is a highly effective scavenger of ·OH radical and stimulate expression of antioxidant enzymes	Free radical scavenger	248, 249
Resveratrol	Resveratrol, a polyphenol compound reported with antioxidant and anti-carcinogenic effect. It can reduce radiation-induced chromosomal aberration in mouse bone marrow cells, when administered (100 mg/kg BW daily) 2 days prior to 3Gy whole body γ-radiation. Moreover, resveratrol is an effective agent in protecting against radiation injury by targeting Sirt1.	It can protect radiation-induced chromosomal aberration	250, 251
EUK (SOD mimetics)	Two lead compounds (synthetic SOD/catalase mimetics), EUK-207 at a dose of 30µM and EUK-451 at a dose of 10µM, exhibited low toxicity and mitigated radiation-induced apoptosis against 2-20Gy. EUK-207 has also been shown to mitigate radiation-induced skin injury and promotes wound healing, when administered 48 h after exposure.	Radio-mitigator	252-254
Carnosine	Carnosine is a dipeptide has antioxidant and anti-inflammatory properties. It has potential to ameliorate irradiation-induced lung injury and radio-protective effect on the wound healing in rats.	It protect from radiation induced lung injury	255, 256
IL-12 (HemaMax)	IL-12 has potential for protection of hematopoietic system, which can attenuate severe myelosuppresion caused by lethal or sub-lethal irradiation. It also have an anti-tumor and anti-angiogenic ability and may have clinical significance in cancer treatment and BMT.	Protection against severe myelosuppresion	257, 258
